# Central 5-HTR2C in the Control of Metabolic Homeostasis

**DOI:** 10.3389/fendo.2021.694204

**Published:** 2021-07-21

**Authors:** Ting Yao, Jiehui He, Zhicheng Cui, Ruwen Wang, Kaixuan Bao, Yiru Huang, Ru Wang, Tiemin Liu

**Affiliations:** ^1^ School of Kinesiology, Shanghai University of Sport, Shanghai, China; ^2^ Department of Physiology and Pathophysiology, School of Basic Medical Sciences, Xi’an Jiaotong University School of Medicine, Xi’an, China; ^3^ School of Life Sciences, Fudan University, Shanghai, China; ^4^ Human Phenome Institute, Fudan University, Shanghai, China; ^5^ State Key Laboratory of Genetic Engineering, Fudan University, Shanghai, China; ^6^ State Key Laboratory of Pharmaceutical Biotechnology, Nanjing University, Nanjing, China

**Keywords:** 5-HTR2C, feeding behavior, glucose homeostasis, obesity, hypothalamus, neural network, energy metabolism, lorcaserin

## Abstract

The 5-hydroxytryptamine 2C receptor (5-HTR2C) is a class G protein-coupled receptor (GPCR) enriched in the hypothalamus and the brain stem, where it has been shown to regulate energy homeostasis, including feeding and glucose metabolism. Accordingly, 5-HTR2C has been the target of several anti-obesity drugs, though the associated side effects greatly curbed their clinical applications. Dissecting the specific neural circuits of 5-HTR2C-expressing neurons and the detailed molecular pathways of 5-HTR2C signaling in metabolic regulation will help to develop better therapeutic strategies towards metabolic disorders. In this review, we introduced the regulatory role of 5-HTR2C in feeding behavior and glucose metabolism, with particular focus on the molecular pathways, neural network, and its interaction with other metabolic hormones, such as leptin, ghrelin, insulin, and estrogens. Moreover, the latest progress in the clinical research on 5-HTR2C agonists was also discussed.

## Introduction

Serotonin, or 5-hydroxytryptamine (5-HT), is an essential neurotransmitter that has been shown to be involved in the regulation of multiple physiological and behavioral functions, including emotion, cognition, sleep, exercise, and energy homeostasis (1, 2). There are seven classes of receptors in the 5-HT family, most of which are G-protein coupled receptors (3, 4). Among them, 5-HTR2C has been shown as a key regulator for feeding and glucose homeostasis. Knock-out of 5-HTR2C in mice resulted in increased food intake, insulin resistance, and obesity (5, 6), while pharmacological activation of the 5-HTR2C inhibits food intake (7). Thus, 5-HTR2C has become a hot target for anti-obesity treatment. For example, the non-selective 5-HTR2C agonist D-Fenfluramine (d-Fen) (**Table 2**), and selective 5-HTR2C agonist lorcaserin (**Table 2**) were approved by Food and Drug Administration for body weight management. However, they were withdrawn due to associated side effects. A better understanding of the mechanism of 5-HTR2C on energy homeostasis will facilitate the development of improved drugs targeting 5-HTR2C pathways for metabolic diseases. In this review, we recapped the molecular mechanisms and discussed the neural circuits of 5-HTR2C in regulating energy metabolism. In addition, the functional interactions between 5-HTR2C and other appetite-regulatory signaling pathways were discussed. Since 5-HTR2C has become one of the most promising targets for treating obesity, we also discussed the clinical application of 5-HTR2C as a potential therapeutic target in treating metabolic diseases.

## 5-HTR2C Signal Transduction

5-HTR2C is one of the first sequenced and cloned 5-HT receptors (8). The gene coding 5-HTR2C is located at chromosome Xq24 in humans. It contains three introns (instead of two, such as 5-HTR2A and 5-HTR2B) and encodes a protein product with seven transmembrane regions. There is more than 80% homology of 5-HTR2C in the transmembrane regions among mice, rats, and humans (9). 5-HTR2C is widely expressed in the central nervous system (CNS) compared to the peripheral nervous system (10). In the CNS, 5-HTR2C is expressed in these known brain areas that are related to energy metabolism, including the ventral tegmental area (VTA), the arcuate nucleus (ARC), the nucleus tractus solitarius (NTS), paraventricular nucleus of the hypothalamus (PVN), and lateral parabrachial nucleus (LPBN) (11). Genetic studies with loss or gain function of 5-HTR2C indicate a key role of 5-HTR2C in these brain regions in maintaining energy homeostasis (12). Pharmacological studies using its agonists or antagonists also revealed that central 5- HTR2C is involved in various metabolic diseases such as diabetes and obesity (13). In line, a higher density of 5-HTR2C was found in the hypothalamus in Prader-Willi syndrome (PWS) patients showing hyper appetite and obesity (14).

**Figure 1:**
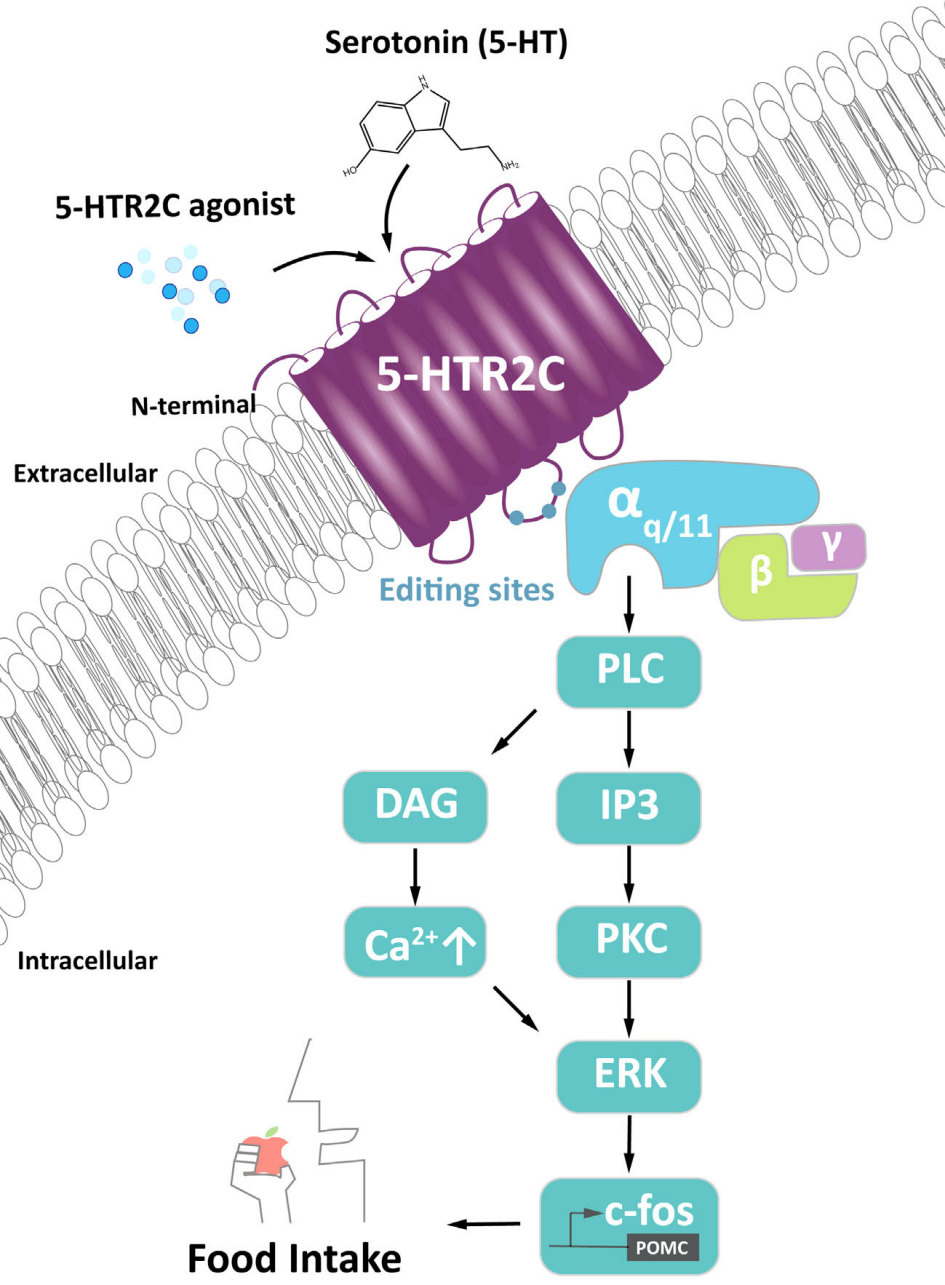
Signaling of 5-HTR2C in POMC neurons generating food intake. Activation of α_q/11_ promotes phospholipase C (PLC) to produce diacylglycerol (DAG) and inositol-1,4,5-triphosphate (IP3). IP3 promotes release of intracellular calcium (Ca^2+^) while DAG binds to downstream effector protein kinase C (PKC), both of which activates c*-Fos via the* extracellular regulated kinase pathway (ERK). C-*Fos* turns on the pro-opiomelanocortin (POMC) promoter, which signals to neurons regulating the food intake signal. This signal is also regulated by 5-HTR2C agonist and serotonin.

The best understood function of 5-HTR2C is food intake regulation through 5-HTR2C action in pro-opiomelanocortin (POMC) neurons in the hypothalamus (**Figure 1**). Serotonin binding to 5-HTR2C leads to the dissociation of a heterotrimeric G protein that binds to the second intracellular loop of 5-HTR2C. Upon dissociation, the subunit G_α/q11_, activates phospholipase C, generating inositol triphosphate (IP3) and diacylglycerol (DAG), which activates Protein Kinase C (PKC). PKC activates the extracellular regulated kinase (ERK) pathway, leading to the phosphorylation of c-*Fos* and POMC synthesis. POMC is processed into α-melanocyte-stimulating hormone (α-MSH) that activates neurons in the PVN *via* melanocortin 4 receptors (MC4Rs) (15). Activation of PVN neurons induces satiety, i.e., cessation of eating, an anorexic response.

Of note, 5-HTR2C is the only known GPCR whose mRNA undergoes post-transcriptional editing to yield different receptor isoforms (16). This RNA editing process further modulates the basal activity of 5-HTR2C and/or the sensitivity of 5-HTR2C (17). The 5-HTR2C mRNA is edited in 5 distinct sites (18) resulting in at least 33 distinct mRNAs and 25 distinct isoforms of the protein in humans (19). The activity of the 5-HTR2C is regulated through the ratio of these truncated to full-length receptors. An increase in the truncated receptor sequesters the full-length receptor intracellularly, decreasing 5-HTR2C signaling (20). Overexpression of fully-edited receptors decreased the expression of POMC in the hypothalamus and caused hyperphagia in mice (21). In addition, mutation of SNORD115, a small RNA that regulates alternative splicing of 5-HTR2C, is observed in most in PWS patients who are characterised by hyperphagia and obesity (14).

Taken together, 5-HTR2C is associated with multiple signal transduction pathways, mobilizing various intracellular signaling molecules. An in-depth understanding of the gene-editing processes of 5-HTR2C in the central regulation of metabolism may help to identify the differentially expressed targets for pharmacological operations and the development of new drugs.

## Feeding Behavior

### 5-HTR2C in POMC Neurons in the ARC and NTS

**Figure 2:**
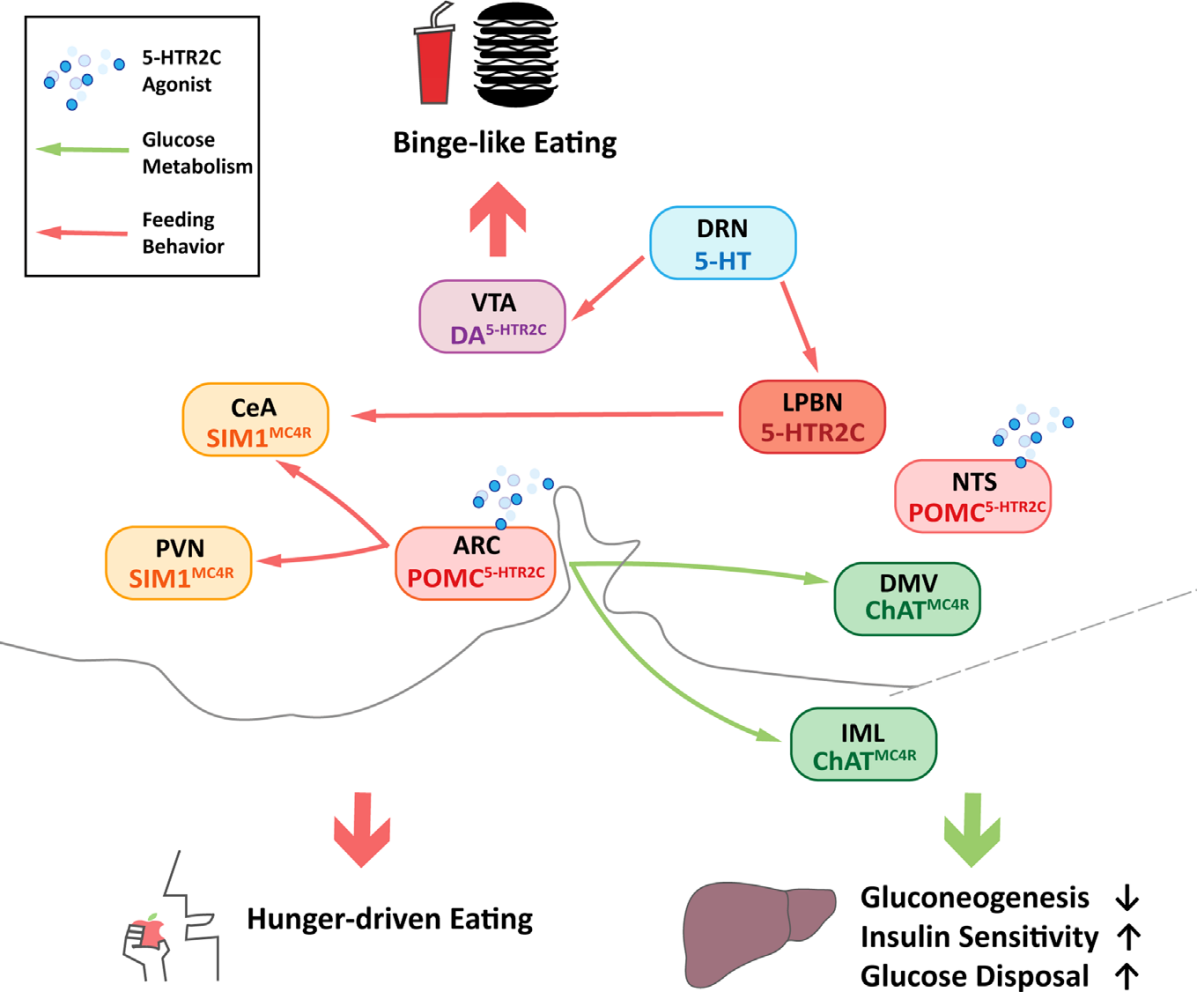
Central neuronal circuits of 5-HTR2C that regulate feeding behavior and glucose homeostasis. Central Nervous System (CNS) 5-hydroxytryptamine receptor 2C (5-HTR2C) may regulate energy metabolism through neuronal circuits. Red arrows designate circuits that regulate three types of feeding behavior which are binge-like eating, sodium intake and hunger-driven eating, while the green arrows show the circuits that regulate glucose homeostasis by reducing gluconeogenesis, increasing insulin sensitivity and glucose disposal. Pink boxes indicate nuclei containing POMC neurons that co-express 5-HTR2C (POMC^5-HTR2C^); Green boxes indicate nuclei containing cholinergic neurons that co-express melanocortin 4 receptors (ChAT^MC4R^); Yellow boxes indicate nuclei containing single minded-1 (SIM1) neurons that co-express melanocortin 4 receptors (SIM1^MC4R^); Light purple box indicates nuclei containing DA neurons that co-express 5-HTR2C (DA^5-HTR2C^); Light blue box indicates nuclei containing 5-HT neurons and orange box indicates nuclei containing a subset of neurons expressing 5-HTR2C. ARC, arcuate nucleus; NTS, nucleus tractus solitarius; PVN, paraventricular nucleus of the hypothalamus; CeA, central amygdala; LPBN, lateral parabrachial nucleus; DRN, dorsal raphe nucleus; VTA, ventral tegmental area; DMV, dorsal motor nucleus of the vagus; IML, intermediolateral nucleus.

POMC neurons in the ARC are characterized as the first-order neurons that regulate energy balance in the hypothalamus (22). In the ARC, most of POMC neurons co-express 5-HTR2C (23) and receive inputs from serotoninergic nerve fibers terminate (24). 5-HTR2C has also been proved to regulate energy homeostasis. Mice with global mutation or knock-out of the *5-HTR2C* gene (2C-null) developed hyperphagia and obesity (**Table 1**) (5, 25, 26), and 5-HTR2C agonist d-Fen was reported to suppress mice food intake, contributing to the anorexigenic effects (27). Electrophysiological studies showed that selective 5-HTR2C agonists, including m-chlorophenyl piperazine (mCPP), d-Fen (23), could activate ARC POMC neurons and stimulate POMC expression by increasing the mRNA level (28–30). ARC POMC neurons produce α-MSH, an endogenous agonist of MC4Rs (31–33). It was reported that the mutation of the *MC4R* gene led to insensitivity to the anorectic effect of d-Fen (34), suggesting that the function of ARC 5-HTR2C required a central melanocortin pathway. In particular, the involved MC4R population was probably located at the single minded-1 (SIM1) neurons in the PVN, because the restoration of MC4Rs in SIM1 neurons in MC4R KO mice was sufficient to rescue anorexigenic effects caused by the 5-HTR2C agonist (32, 35, 36). Moreover, deleting the 5-*HTR2C* gene only in POMC neurons (POMC-2C-null) increased mice food intake (**Table 1**) (15), while re-expressed 5-HTR2C in POMC neurons (POMC-2C-RE) could rescue this phenotype (**Table 1**) (25). Further, at the cellular level, transient receptor potential cation 5 (TrpC5) was required to activate POMC neurons by 5-HTR2C, as the intraperitoneal injections of 5-HTR2C agonist failed to suppress food intake in *TrpC5* KO mice (37). Thus, the feeding inhibitory effect by activating 5-HTR2C was at least partly mediated by the POMC neurons in the ARC (**Figure 2**) (23, 38).

**Table 1:** Phenotypes of the 5-HTR2C deficient mice.

Mice Model	Body Weight	Fat Mass	Lean Mass	Food Intake	Binge-like Eating	Hepatic Glucose Production	Reference
2C-null	**↑**	**↑**	**↓**	**↑**	**↓**	**↑**	(5, 25)
POMC-2C-null	**↑**	**↑**	**↓**	**↑**	**↓**	**↑**	(15)
POMC-2C-RE	**↔**	**↔**	**↔**	**↔**	**/**	**↔**	(25)
DA-2C-RE	**/**	**/**	**/**	**↑**	**↔**	**/**	(15)
DA-2C-KO	**/**	**/**	**/**	**↑**	**↓**	**/**	(15)

‘↑’, Increased; ‘↓’, Reduced; ‘↔’, No change; ‘/’, Unknown. 2C-null is a loxed transcription blocker (loxTB) 5-HTR2C mouse line lacking functional 5-HTR2C globally; POMC-2C-null mice with previously characterized animals in which cre is constitutively (developmentally) expressed in POMC neurons to ablate 5-HTR2C specifically; POMC-2C-RE mice with 5-HTR2C re-expressed specifically and only in POMC neurons; DA-2C-RE mice with the expression of endogenous 5-HTR2C only in DA neurons; DA-2C-KO with deletion of endogenous 5-HTR2C only in DA neurons.

In addition to the ARC, NTS, a brainstem center for satiety signals, also contains substantial POMC-expressing neurons that co-express 5-HTR2C (39, 40). Studies have shown that 5-HT2CR in the NTS is involved in the anorexic effect of the two 5-HT2CR agonists, lorcaserin and WAY161,503 and chemogenetics activation of 5-HTR2C-expressing neurons in the NTS decrease food intake in mice (38). Interestingly, different from ARC POMC neurons, NTS POMC neurons decreased food intake more significantly and rapidly, in other words, 5-HTR2C agonist lorcaserin required a longer time to decrease mice food intake in the ARC as effectively as in the NTS (38, 41). Therefore, NTS POMC neurons appear to mediate the inhibitory effects of lorcaserin on feeding, but the downstream pathway remains elusive (**Figure 2**). Studies had shown that PVN and central amygdala (CeA) could be innervated by NTS POMC neurons (42). Both of them are key brain regions involved in the regulation of feeding behavior, but the roles of these brain regions warrant further investigation.

### 5-HTR2C in Dopamine Neurons in the VTA

Apart from homeostatic feeding, 5-HTR2C is also involved in hedonic feeding behaviors, defined as ingestion of a large amount of food in a short timeframe for pleasure (43, 44). The central dopamine (DA) system has been implicated in the pathophysiology of binge eating (45); 5-HT releasing neurons in the dorsal raphe nucleus (DRN) directly innervate DA neurons in VTA (46). In the VTA, DA neurons were proved to co-express 5-HTR2C (47), suggesting that 5-HTR2C probably interacted with VTA DA neurons to regulate binge eating. Moreover, intraperitoneal injections of 5-HTR2C agonists significantly suppressed binge-like eating in wild-type mice, while the 2C-null mice showed no effect (48). Indeed, specific knock-out of *5-HTR2C* gene in the VTA DA neurons (DA-VTA-KO) blunted the suppression of binge-like eating by 5-HTR2C agonist (**Table 1**) (48). These observations indicate that 5-HTR2C can act downstream the DRN 5-HT neurons to inhibit food intake. However, the feeding control by VTA DA 5-HTR2C-expressing neurons seemed specific to hedonic rather than hunger-driven eating, as re-expression of 5-HTR2C in DA neurons (DA-2C-RE) did not affect normal food intake in mice even when administered with the 5-HTR2C agonist (**Table 1**) (48). But the downstream neural circuits of the DA 5-HTR2C-expressing neurons still remain unclear. Studies have found that administration of cocaine can increase DA releases in the nucleus accumbens (NAc), an effect that can be blocked by local injections of 5-HTR2C agonist in the VTA (49). Given the abundant connectivity between VTA neurons and the NAc, it would be interesting to know that DA 5-HTR2C neurons in the VTA regulate binge eating by projecting to the NAc (**Figure 2**).

### 5-HTR2C in the LPBN on Sodium Intake

Sodium ions are important minerals for maintaining extracellular fluid and blood volume (50). Studies have found clues for 5-HTR2C in the LPBN to regulate sodium appetite (51). Ingestion of high-concentration sodium-containing food increased c-*Fos* expression in neurons that co-express 5-HTR2C in the LPBN (52–54). Furthermore, specific activation of LPBN 5-HTR2C neurons rapidly suppressed sodium intake in mice. By contrast, inhibition of the 5-HTR2C neurons of LPBN increased the intake of sodium-containing foods (55). Furthermore, electrophysiological studies suggested an abundant connectivity between LPBN 5-HTR2C neurons and CeA (**Figure 2**) (54). In vivo optogenetics stimulation further indicated that LPBN 5-HTR2C neurons could suppress sodium appetite *via* projections to CeA (54). Moreover, raphe nuclei probably modulate the neurons in the LPBN through serotoninergic projections. The injection of retrobeads into the LPBN of wildtype mice showed co-localization of 5-HT and retro bead-labeled cells in the DRN and the median raphe nucleus (MnR) (54). In conclusion, LPBN 5-HTR2C neurons may receive 5-HT signals from median raphe nucleus (MRN)/DRN and project to the CeA to regulate sodium intake (**Figure 2**).

## Energy Expenditure

The 5-HT signaling pathway is closely related to individual energy storage and expenditure. Inhibiting the 5-HT signaling pathway can increase individual thermogenesis in mice (56). Studies found that the knock-out of 5-HTR2C affected the activity level and energy expenditure in mice. The mutant mice exhibited hyperactivity, and increased total energy expenditure, while reducing energy expenditure during exercise. At nine months old, elevated mRNA levels of uncoupling protein 2 (UCP2) were detected in the liver, skeletal muscle, and white adipose tissue of the mutant mice (57). The mice targeted restoration of POMC only within 5-HTR2C expressing cells showed sex differences in physical activity, energy expenditure, and the development of obesity (58). In addition, mutation of the 5-HTR2C gene can increase the mRNA level of UCP1 in brown adipose tissues and reduce fat accumulation in mice (59–61). In summary, 5-HTR2C can affect the energy expenditure of tissues or individuals in diverse ways.

## Glucose Homeostasis

In addition to regulating food intake, serotonin is essential in regulating glucose homeostasis. 5-HT produced by enterochromaffin cells in the gut can act as an paracrine signal modulating islet β cell activity and proliferation (62, 63). It has been shown that 5-HTR2B agonists could promote insulin secretion (64). Meanwhile, studies have revealed the role of 5-HTR2C in the POMC neurons in mediating blood glucose, suggesting a central role of 5-HTR2C in glucose metabolism. Indeed, 2C-null mice manifested insulin resistance (15, 25), and POMC-2C-RE mice was sufficient to rescue the impairment (**Table 1**) (6). On the other hand, 5-HTR2C agonist lorcaserin could significantly improve glucose and insulin tolerance in wild-type mice, and these effects were abolished in *POMC* gene deficient mice (POMC-NEO) and restored in POMC-2C-RE mice (65). Studies showed that the glycemic effect of 5-HTR2C in POMC neurons was mediated by cholinergic (ChAT) MC4Rs in dorsal motor nucleus of the vagus (DMV) and the intermediolateral nucleus (IML) (32, 65, 66), which was different from the forebrain SIM1 MC4Rs implicated in feeding behavior, indicating the subsets of POMC 5-HTR2C neurons in controlling feeding behavior and glucose homeostasis might be different (**Figure 2**). It was shown that 5-HTR2C agonist m-CPP and lorcaserin can improve glycemic control independently of body weight (6, 15, 65, 67). The improved glucose tolerance in mice by lorcaserin was found to be mediated by reducing the hepatic glucose production and improving glucose disposal, without change of insulin secretion (65) (**Table 1** and **Figure 2**). Interestingly, a recent study showed that a subset of POMC neurons may have the ability to promote hepatic glucose production, which was speculated to be relevant with the heterogeneity of POMC neurons (68). Given the complex functions of POMC neurons in the brain, the relationship between this subset of POMC neurons and POMC 5-HTR2C neurons remains to be further elucidated. In analyzing the heterogeneity of different parts of the same tissue, the spatial transcriptomics studies may be helpful. Studies had preliminarily used the spatial transcriptome to reveal the heterogeneity of tumor tissue (69). Integrating the transcriptome profiles and projection patterns of individual neurons may help to clarify how POMC 5-HTR2C neurons process various stimuli at the single-neuron level.

## Interaction of 5-HTR2C With Bodyweight Regulatory Signals

### Leptin

Leptin, a key regulator for the metabolism, is secreted from adipocytes (70). It prevents bodyweight gain by suppressing feeding and increasing energy expenditure (71). Mutations of the gene encoding leptin in mice (*ob/ob*) lead to severe obesity and increased appetite (72). The leptin receptor (LepR) mediates the effects of leptin on body weight, and it is involved in the majority of leptin’s actions in the brain (73). Double fluorescent *in situ* hybridization experiments showed that in the hypothalamic, the neurons which express LepR also co-express 5-HTR2C, including the ARC and the ventromedial hypothalamus (VMH) (74, 75). However, selective knock-out of LepR in 5-HTR2C-expressed neurons exhibited neither hyperglycemia nor alteration in serum insulin or leptin concentrations (76). Further, single-cell transcriptomic data showed that the LepR-expressing POMC cells formed a molecularly distinct cluster relative to POMC neurons expressing the 5-HTR2C (77, 78), indicating that leptin probably affected systemic energy balance through different POMC neuronal subsets. In particular, Daniel D et al. clarified that brain 5-HT neurons did not express LepR and therefore not directly responded to leptin (79). In conclusion, whether the leptin signaling pathway interacts with 5-HT remains controversial, which needs further research.

### Ghrelin

Ghrelin is a stomach-derived body weight regulatory signal stimulating feeding *via* the growth hormone secretagogue receptors (GHSRs). The ghrelin signaling pathway interacts with 5-HT. The appetite-stimulating activity of ghrelin is shown to be mediated by the inhibition of serotonin release (80). GHSRs in ARC is expressed in 94% of nerve peptide Y (NPY) neurons and 8% of POMC neurons (81), and is co-localized with 5-HTR2C neurons. Studies have shown that the 5-HTR2C is dimerized with the GHSRs to inhibit its orexigenic activity (82). The activation of 5-HTR2B and 5-HTR2C reduced the gastric and hypothalamic secretion of ghrelin (83). 5-HTR2C agonist like lorcaserin inhibits the increase of plasma ghrelin level induced by fasting. Besides, 5‐HTR2C antagonism reduces dimerization and increases GHSR‐induced food intake, indicating that 5-HTR2C can change the regulation of ghrelin on feeding (84, 85). Overall, 5‐HTR2C and its interaction with GHSR are probably a valuable target for designing new compounds to prevent obesity.

### Insulin

Insulin efficiently crosses the blood-brain barrier *via* receptor-mediated transport (86). Besides, the insulin receptor is widely expressed in the CNS, including the cerebral cortex, hippocampus, and hypothalamus (87). Mice with targeted mutation in the 5-HTR2C gene resulted in insulin resistance and type 2 diabetes (T2D), with antecedent hyperphagia and obesity (26, 88), suggesting an interaction of insulin with 5-HTR2C on energy metabolism. Infusion of insulin in the hypothalamic could briefly enhance 5-HT release in rostromedial hypothalamus (89), and systemic administration of 5-HTR2C agonist mCPP by osmotic minipumps could reduce fasting plasma insulin level through POMC neurons in diet-induced obesity (DIO) mice without altering blood glucose (90). However, single-cell transcriptomic data showed the subset of POMC neurons that expressing 5-HTR2C and insulin receptor were not the same (77), which should be further investigated.

### Estrogens

The gene encoding 5-HTR2C has been mapped to human chromosome X, suggesting a sex-dependent role for 5-HTR2C signaling (91). When 5-HTR2C agonists and antagonists were used in elderly mice exposed to stress, different feeding phenotypes were found in females and males (92). When food is reduced due to stress, the female mice recovered more quickly than the male mice (92). In aged male mice, exposure to novelty stress promoted 5-HTR2C protein synthesis in PVN stress-specific neurons and activated neurons that expressed 5-HTR2C (93). In contrast, there was no change in 5-HTR2C and *c-Fos* co-positive cell counts in the PVN of aged female mice exposed to stress (93). It was unclear if these sex differences were due to gonadal hormones or the organizational effect, but estradiol was reported to enhance 5-HTR2C protein synthesis in the DRN region (94), caudal brainstem, and hypothalamus (93, 95).

### Cholecystokinin

As a bodyweight regulatory signal, cholecystokinin(CCK), secreted by the gastrointestinal tract and neurons in brain, stimulates satiety and suppresses feeding behavior (96, 97). Some studies have found that CCK can act synergistically with 5-HT to inhibit food intake by simultaneously activating CCK-1 and 5-HTR3A (98, 99), and 5-HTR1A are also involved in CCK induced anorexic behavior (100, 101). However, there is little research on the association between 5-HTR2C and CCK signaling in CNS (102), which is probably a direction for future research.

### NPY/AgRP

Studies have confirmed co-expression of NPY and 5-HTR2C in the lateral hypothalamus, the basolateral nucleus and ARC (103–105). Intraperitoneal injection with 5-HTR2C agonist lorcaserin could significantly reduce the expression of NPY mRNA in the ARC, while 5-HTR2C antagonist risperidone caused the opposite effect (103). In addition, injection of 5-HTR2A/2C agonist 1-(2,5-dimethoxy-4-iodophenyl)-2-aminopropane (DOI) into the PVN, but not the perifornical hypothalamus and VMH, could suppress NPY-induced feeding behavior (106, 107). In summary, the effect of 5-HTR2C on feeding seems to be highly associated with NPY/AgRP signaling, and more research on how 5-HTR2C affects NPY/AgRP neurons is required for further investigation.

## Clinical Application

### Obesity

Obesity prevalence calls for new methods of appetite suppression and weight loss. Satiety and appetite control pathways have been widely studied in animals and humans, but the exact underlying molecular mechanism is still unclear (108, 109). Nowadays, some drugs used to treat obesity have side effects. At present, 5-HTR2C is one of the most promising targets for new weight-loss drugs. Many modulators targeting 5-HT signaling, including sibutramine (serotonin and adrenaline reuptake inhibitors) (**Table 2**), mCPP, and fenfluramine (also named as fluoxetine, selective serotonin reuptake inhibitors) (**Table 2**) have been used as appetite suppressants (27, 110, 111). Sibutramine and fluoxetine can increase extracellular serotonin levels *in vivo*, non-selectively stimulate all postsynaptic subtypes, and then stimulate 5-HTR2C to suppress food intake (112, 113). Heisler (114) reported that mCPP did not inhibit food intake in 5-HTR2C knock-out mice and weakened the swallowing effect of fenfluramine (serotonin releasing agent and reuptake inhibitor), demonstrating the key role of 5-HTR2C in satiety induction by d-fenfluramine. Fenfluramine, an effective treatment for obesity, sold as Pondimin ^®^/Redux ^®^, reduces appetite. Fenfluramine binds weakly to the serotonin 5-HTR2C, d-Fen binds to and activates the serotonin 5-HTR2B and 5-HTR2C with high affinity and the serotonin 5-HTR2A with moderate affinity (115–117). However, fenfluramine is associated with side effects of valvular heart disease and pulmonary hypertension, prompting it to withdraw from clinical use (118, 119).

**Table 2:** 5-HTR2C related drugs in this review.

Name	Mechanism of Action	Side Effect	Application
Lorcaserin	selective 5-HTR2C agonist	headache, fatigue, nausea, dry mouth, and constipation	weight-loss drug
D-Fen	serotonin releasing agent and reuptake inhibitor	cardiac complications	weight-loss drug
Sibutramine	serotonin and adrenaline reuptake inhibitor	stroke, myocardial infarction	weight-loss drug
Fluoxetine	selective serotonin reuptake inhibitor	anorexia	an approved drug to treat depression and obsessive-compulsive disorder
m-CPP	agonist of 5-HTR2C and 5-HTR1B	anxiety, negative mood measured	decrease food intake and enhance microstructural measures of satiety

Lorcaserin is an effective and selective 5-HTR2C agonist that reduces food intake and body weight in rodents in a dose-dependent manner (120). Lorcaserin was approved by Food and Drug Administration for weight management in adults with body mass index (BMI) ≥ 30 kg/m^2^ or BMI ≥ 27 kg/m^2^ with at least one weight-related complication. Since 2013, lorcaserin has been sold in the United States under the name of Belviq ^®^. The safety and efficacy of lorcaserin have been determined by three phases III clinical trials, one cardiovascular (CV) outcome trial, and four randomized controlled trials (121–123). Animal experiments showed that after 28 days of treatment in diet-induced obesity rats, there was no aortic and mitral regurgitation in any treatment group (124). A 6-month randomized, placebo-controlled, double-blind clinical trial also found that lorcaserin could reduce weight and improve cardiac metabolic risk factors in obese adults, thus modifying circulating body weight regulatory signals associated with energy balance and decreasing the risk of cardiovascular disease (125). Recent follow-up data have shown that the drug probably increases cancer risk, and further research is needed. The role of 5-HTR2C in POMC neurons and the new role in neural circuits suggest that the new anti-obesity drugs act directly on the CNS, thereby reducing the negative effects caused by acting on the periphery, which will be discussed in the future.

### Diabetes

As a chronic disease, the prevalence of T2D continues to rise worldwide, highlighting the clinical need for a variety of treatment options. The current first-line drugs for T2D target peripheral tissue to improve blood glucose and insulin function (126, 127). 5-HTR2C has been found to regulate glucose homeostasis during weight loss, which is expected to become a candidate target (128, 129) for the treatment of diabetes. Yuan et al. discovered that the-759C/T polymorphism of the *5-HTR2C* gene was associated with obesity and T2D (130). The lower frequency of-759T allele in the *5-HTR2C* gene was associated with T2D but not associated with obesity in men and women (131), resulting from alleles type from promoter activity and transcriptional level, thus preventing the development of T2D.

A retrospective analysis of the Phase III BLOOM-DM study showed that lorcaserin combined with diet and exercise decreased blood glucose within 2 weeks (132–134). In the study of the effect of lorcaserin on weight loss in patients with T2D, lorcaserin could also decrease the Hemoglobin A1c of diabetic patients, providing direct evidence support for the treatment of diabetes. Besides, reducing fasting plasma glucose and Hemoglobin A1c was greater in people with no significant weight loss, suggesting that it could benefit blood sugar independent of weight loss. Besides, more clinical studies are needed to demonstrate this regulation in the future.

### Cardiovascular System

Obesity and metabolic syndrome can increase the risk of cardiovascular disease. Weight-loss drugs can affect cardiovascular health by losing weight and directly acting on the cardiovascular system (135, 136). It was found that subcutaneous injection of 5-HTR2C agonist mCPP (3 mg/kg) had no significant effect on heart rate and meant arterial blood pressure (137). Lorcaserin, a selective 5-HTR2C agonist, did not seem to have a negative effect on the cardiovascular system at very high concentrations (125). Alpana P Shukla et al. (7) summarized the pharmacodynamic and pharmacokinetic characteristics of lorcaserin and discussed efficacy and safety data from major clinical trials. The bodyweight could be reduced by a certain dose of treatment. Therefore, the cardiac metabolic parameters could be significantly improved. In the CAMELLIA-TIMI 61 trial, the incidence of adverse cardiovascular events and conversion to T2D in obese and overweight subjects with cardiovascular disease or multiple cardiovascular risk factors was assessed. It was concluded that the safety of the drug could be guaranteed.

In conclusion, as the first selective 5-HTR2C agonist approved for human weight control, the 5-HTR2C agonist lorcaserin has been widely used in clinical and scientific research after being launched in 2012. Although CAMELLIA-TIMI 61 research found no significant difference in cancer incidence during the first few months of treatment, the imbalance increased with the duration of lorcaserin, suggesting that the drug increased the risk of cancer. The cardiovascular effects of other anti-obesity drugs like liraglutide, bupropion/naltrexone, and phentermine/topiramate remain uncertain (138). Due to the side effects of drugs, there is no better drug to treat obesity. Although 5-HTR2C is the target of several anti-obesity drugs, its side effects limit their clinical application. However, the specific neural circuits of 5-HTR2C expressing neurons and the detailed molecular pathways of 5-HTR2C signaling on metabolic regulation will help to develop better treatment strategies for metabolic disorders. To solve the side effects caused by other drugs on the peripheral spectrum, future drugs that target the CNS will give us more inspiration.

## Discussion

In summary, the action of CNS 5-HTR2C neuron contributes to the regulation of energy homeostasis and have greatly advanced the understanding of the physiology and behavioral functions of 5-HTR2C in the brain. However, there are many questions. As a GPCR, understanding the *5-HTR2C* gene-editing processes is helpful to study the weight-loss drug. However, the detailed molecular mechanisms remain unclear. Furthermore, there is growing interest in brain control of metabolism. Here, we summarized the 5-HTR2C related metabolic circuit of feeding behavior and glucose homeostasis in the brain, and we found that the mechanism of 5-HTR2C in the central cortex still needs to be further clarified. To explore the systemic effects of 5-HTR2C, we also discussed the relationship between metabolic hormones and 5-HTR2C. From the perspective of clinical application, the functions of weight-loss drugs now are mostly concentrated on systemic administration, resulting in negative effects. In the future, 5-HTR2C in the brain may become a potential for the treatment of obesity and type 2 diabetes.

## Author Contribution

JH, ZC, and RWW conceived and wrote the manuscript. KB modified the figure drawing. YH revised the overall framework of the article. TY, RW, and TL were responsible for the article structure, scientific logic, and innovative examination of the overall article. All authors contributed to the article and approved the submitted version.

## Funding

The study was funded by a grant from the National Key Research and Development Program of China (2019YFA0801900, 2018YFA0800300), the National Natural Science Foundation of China (31971074), and the open fund of state key laboratory of Pharmaceutical Biotechnology, Nan-jing University, China (KF-GN-201701).

## Conflict of Interest

The authors declare that the research was conducted in the absence of any commercial or financial relationships that could be construed as a potential conflict of interest.
